# Biological factors influencing depression in later life: role of aging processes and treatment implications

**DOI:** 10.1038/s41398-023-02464-9

**Published:** 2023-05-10

**Authors:** Sarah M. Szymkowicz, Andrew R. Gerlach, Damek Homiack, Warren D. Taylor

**Affiliations:** 1 Center for Cognitive Medicine, Department of Psychiatry and Behavioral Science, Vanderbilt University Medical Center, Nashville, TN USA; 2 Department of Psychiatry, University of Pittsburgh, Pittsburgh, PA USA; 3 Department of Psychiatry, University of Illinois-Chicago, Chicago, IL USA; 4 Geriatric Research, Education, and Clinical Center, Veterans Affairs Tennessee Valley Health System, Nashville, TN USA

**Keywords:** Depression, Pathogenesis, Prognostic markers

## Abstract

Late-life depression occurring in older adults is common, recurrent, and malignant. It is characterized by affective symptoms, but also cognitive decline, medical comorbidity, and physical disability. This behavioral and cognitive presentation results from altered function of discrete functional brain networks and circuits. A wide range of factors across the lifespan contributes to fragility and vulnerability of those networks to dysfunction. In many cases, these factors occur earlier in life and contribute to adolescent or earlier adulthood depressive episodes, where the onset was related to adverse childhood events, maladaptive personality traits, reproductive events, or other factors. Other individuals exhibit a later-life onset characterized by medical comorbidity, pro-inflammatory processes, cerebrovascular disease, or developing neurodegenerative processes. These later-life processes may not only lead to vulnerability to the affective symptoms, but also contribute to the comorbid cognitive and physical symptoms. Importantly, repeated depressive episodes themselves may accelerate the aging process by shifting allostatic processes to dysfunctional states and increasing allostatic load through the hypothalamic–pituitary–adrenal axis and inflammatory processes. Over time, this may accelerate the path of biological aging, leading to greater brain atrophy, cognitive decline, and the development of physical decline and frailty. It is unclear whether successful treatment of depression and avoidance of recurrent episodes would shift biological aging processes back towards a more normative trajectory. However, current antidepressant treatments exhibit good efficacy for older adults, including pharmacotherapy, neuromodulation, and psychotherapy, with recent work in these areas providing new guidance on optimal treatment approaches. Moreover, there is a host of nonpharmacological treatment approaches being examined that take advantage of resiliency factors and decrease vulnerability to depression. Thus, while late-life depression is a recurrent yet highly heterogeneous disorder, better phenotypic characterization provides opportunities to better utilize a range of nonspecific and targeted interventions that can promote recovery, resilience, and maintenance of remission.

## Introduction

Late-life depression (LLD) is major depressive disorder (MDD) occurring in adults age 60 years or older [1]. It is common, with ~5% of community-dwelling elders meeting DSM5 diagnostic criteria [2] and 10–16% of older adults exhibiting clinically significant depressive symptoms that may not meet full criteria [2, 3]. LLD is a malignant illness that increases disability [4], contributes to poorer medical outcomes [5], and is associated with increased suicide risk and mortality [6, 7].

LLD is further characterized by poor or impaired cognitive performance. Reduced executive functioning is common, affecting verbal fluency, response inhibition, set-shifting, working memory, and problem-solving [8]. Individuals with LLD also exhibit poor performance in other cognitive domains, including episodic memory, visuospatial skills, and processing speed [9–11]. Slower processing speed is particularly important, partly mediating impaired performance in other cognitive domains [11–13]. Although cognitive performance improves with successful treatment, deficits typically persist, and older depressed adults have an increased risk of dementia [14].

Such adverse outcomes may be related to LLD’s recurrent nature [15]. LLD is often a recurrent or chronic illness [16], although continuing antidepressant medication during remission reduces recurrence risk [17, 18]. However, even with maintenance treatment, ~35–40% of depressed elders experience recurrence in 2 years, with over 50% experiencing recurrence over four years [16, 18, 19].

Recurrence is particularly relevant for individuals with an initial onset of depression in early- or midlife. These individuals often experience multiple prior depressive episodes and are now in the geriatric age range. Individuals with early-onset LLD, typically defined as occurring before age 50–60 years, exhibit greater residual depression severity over time, more frequent suicidal thoughts, and a greater risk of recurrence following remission [16, 20]. Early-onset depression is characterized by stronger familial history and genetic loading, greater anxiety and reactions to stressful life events, maladaptive personality traits, and hormonal fluctuations associated with early-life reproductive events [21–26]. Individuals with late-onset depression are more often widowed, present with more apathy and somatic symptoms, poorer cognitive performance, greater cognitive decline and medical morbidity, and more severe atrophic and vascular changes on neuroimaging [26–32]. Although useful for clinical characterization, this age of onset dichotomization obfuscates potentially important differences in causal factors that influence the onset or recurrence of depression across the lifespan. Moreover, it does not address a parallel hypothesis that depression itself is toxic, with recurrent episodes increasing the allostatic load or “wear and tear” on the body, contributing to accelerated brain aging and vulnerability to poor longitudinal clinical, cognitive, and medical outcomes [15].

**Fig. 1:**
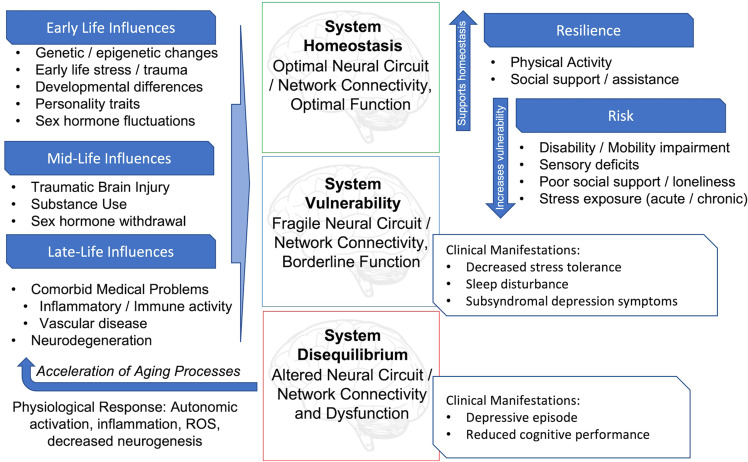
Lifespan model of late-life depression. Symptoms of depression are the behavioral manifestation of increasingly disrupted brain networks. Multiple influences contribute to network fragility and dysfunction across the lifespan, with some being linked to clear developmental periods. These may be additive and cumulative over time, although other risk and resiliency factors may be modifiable and targeted by specific treatments. Unchecked, repeated depressive episodes and their associated physiological responses may have deleterious effects contributing to accelerated aging.

Based on past work [15], we present a model (Fig. 1) where disruption of functional brain network homeostasis contributes first to subclinical depressive symptoms and decreased stress tolerance. If unchecked, this progresses to discrete depressive episodes and reduced cognitive performance. Various biological and environmental factors across the lifespan increase the vulnerability of key networks to disequilibrium, with potentially modifiable behavioral and social factors contributing either to vulnerability or resilience. In turn, depressive episodes alter physiological systems that accelerate aging processes and contribute to adverse longer-term outcomes.

Building on this model, we first present a network-based model of depression. We then focus on etiological influences across the lifespan that increase depression risk, primarily focusing on those salient to later life. Next, we consider depression as a contributor to accelerated aging. Finally, we review treatment for LLD and how interventions may support resilience to future episodes.

## Neural networks implicated in depression

**Fig. 2:**
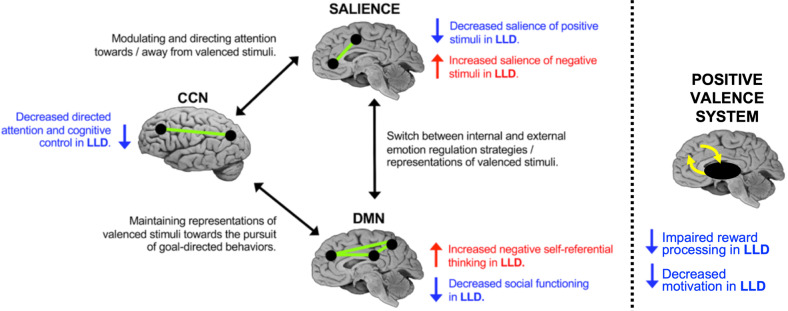
Network model of late-life depression. The model details findings within each intrinsic functional network and between functional networks, specifically the default mode network (DMN), the cognitive control network (CCN), and the anterior salience network (ASN). Disruption in network connectivity influences the cognitive processes, giving risk to the behavioral manifestations of depression. Impaired function of the positive valence system involving the mesolimbic system likely also contributes to depressive behavior [37], although how this system interacts with interacts with intrinsic functional networks in LLD is not entirely clear. The model and figure [34] used with permission. Reprinted from Gunning et al. [34], Copyright 2021, with permission from Elsevier.

Altered neural network function is thought to result in the behavioral manifestations of depression [33]. The triple network model (Fig. 2) [33, 34] posits that depression is related to the aberrant function of the default mode network (DMN), cognitive control network (CCN), and anterior salience network (ASN). Positive valence system circuits involved in reward function are additional contributors [35, 36], although this has received less attention in LLD [37]. These networks likely influence depressive behavior across the lifespan, although the etiological factors contributing to network dysfunction change with aging. Although heterogeneity in LLD prevents sweeping generalizations [38], there is support for this network-centric model [34, 39].

The DMN is implicated in self-referential processes [40] including rumination [41], making it a target of investigation in depression [33, 42]. However, there is little consensus on how the DMN is altered in depression [43]. Early studies reported hyperconnectivity within the DMN relative to healthy controls [42], though recent meta-analyses reported no difference in DMN connectivity [44] or even hypoconnectivity between DMN regions [45]. DMN functional connectivity appears to be altered in LLD [46, 47] and such differences may persist into remission [46].

The CCN is primarily involved in top-down executive functions [48]. Substantial evidence suggests CCN integrity differentiates healthy controls from depressed individuals [42] and influences treatment response [49]. The CCN may be especially relevant in LLD characterized by executive dysfunction [50, 51], where aberrant functional connectivity of the CCN (particularly the dorsolateral prefrontal cortex [DLPFC] hub), is associated with executive deficits [52].

The ASN is implicated in switching and control of attentional processes [53], and ASN dysfunction in depression biases individuals towards negative stimuli and processing [54, 55]. Unlike the DMN, the ASN has proven relatively reliable in distinguishing between healthy controls and depressed individuals [42, 44, 56]. Both structural and functional connectivity of the ASN are reduced in LLD [57].

Alterations in the positive valence system are additive. Conceptually, much work focuses on the dopaminergic mesolimbic pathway, projecting from the ventral tegmental area to the ventral striatum, nucleus accumbens, and medial temporal structures [37]. Dysfunction in this system influences a range of reward functions including valuation, decision-making, effort, and learning [37, 58]. Behaviorally, this contributes to anhedonia, motivational disturbances, and willingness to expend effort [37].

### Neural networks and aging

The connectivity and function of these networks change with age. Cross-sectional and longitudinal studies demonstrate that frontal regions comprising the associative networks described above (plus the dorsal attention network) exhibit reduced intra-network connectivity and increased inter-network connectivity with aging [59–69]. These changes may reflect a decline in network efficiency and/or serve as a compensatory mechanism that maintains normal brain function in context of gray matter loss or white matter degradation [70, 71]. Supporting this latter hypothesis are studies showing increased activation in frontal regions in older adults compared to younger adults that are associated with better cognitive performance with aging [72]. Increased prefrontal activation is further associated with reduced white matter integrity, again supporting compensation [73, 74]. These age-related network changes have been replicated using both structural and functional network measures, further associating aging with lower strength and density of structural connections and decoupling of structural and functional connectivity, particularly in network hubs [63]. This may represent a rerouting of information flow in the brain intended to circumnavigate degraded white matter pathways or avoid regions that have suffered neuronal loss. The ability of the brain to successfully “rewire” during aging may be crucial for maintaining cognitive performance and reflect cognitive reserve [70]. Moreover, age-related changes may contribute to network fragility, increasing risk for LLD. While there is some evidence that network organization properties may differ according to age of onset [57], clear differences in neural network configuration between early- and late-onset LLD have not been identified.

## Factors contributing to depression vulnerability

### Early- and midlife risk factors

Older depressed adults carry the same vulnerabilities that increased risk for depression earlier in life. As the list of potential contributors to MDD risk is beyond this review, we focus on mechanisms of relevance to LLD.

Genetic factors that influence MDD vulnerability likely persist with aging. Genome-wide association studies (GWAS) identified several hundred potential genetic risk variants, including genes involved in synaptic structure and neurotransmission [75]. In order to influence depression risk, such genetic factors would need to directly or indirectly alter brain network function or stability [76]. However, concerns persist about translating these findings to the individual level, both due to the contributions of small-effect polymorphisms that may be missed on GWAS, and due to diagnostic heterogeneity within MDD [77]. What risk genes contribute to depression vulnerability may change across the lifespan, particularly if they affect brain aging. For example, some work supports that vascular risk genes may be germane in LLD [78].

Adverse childhood experiences (ACEs), such as abuse, parental loss, and bullying, are associated with a host of health disorders, including depression [79]. They are also associated with differences on neuroimaging and cognitive testing [80]. ACEs may contribute to depression vulnerability through hypothalamic–pituitary–adrenal (HPA) axis responses leading to increased activity of corticotropic releasing factor neurons [81]. ACEs can further result in epigenetic changes [24] that may increase depression vulnerability by influencing glucocorticoid signaling, serotonergic function, and neurotrophic factors [24]. The relationship between ACEs and depression vulnerability persists into later life [82, 83], where the relationship between ACEs and depression may be mediated by inflammation [82]. Stressful events occurring in adulthood or later life also increase the risk for new depressive episodes and depression persistence [84, 85].

Personality traits are similarly associated with depression. Traits that influence how individuals interact with and respond to their environments originate from variability in functional brain networks [22]. They arise from a complex interplay between brain development, genetic predisposition, and early environmental exposures. Neuroticism, a predisposition to experience psychological distress and negative mood states, is well-studied and shares some conceptual overlap with LLD [86]. Higher levels of neuroticism in LLD are associated with poorer antidepressant response and greater risk of cognitive decline [86–88].

Reproductive events including puberty, menstrual cycling, pregnancy, and menopause are associated with both new-onset and recurrent depression [23]. These events contribute to a higher risk of depression for women than men [89], particularly during reproductive years [90]. These relationships are due to fluctuations of ovarian hormones that influence neurotransmitter function, neuroendocrine processes, and inflammation [91]. Menopause is particularly relevant to aging, with decreased estrogen production potentially reducing its neuroprotective effects [92]. A longer exposure to endogenous estrogens, operationalized as an older age at menopause, is associated with a lower risk of subsequent depression [93], while earlier menopause, including surgically-induced menopause, is associated with cognitive decline and dementia [94].

Other exposures influence MDD, including comorbid mental health disorders, substance misuse, and traumatic brain injury [95]. Medical comorbidity also contributes, including a bidirectional relationship between depression and obesity [96] that may be mediated through immune system activation and inflammation [96]. Obesity increases the risk for other morbidities associated with LLD, including pain syndromes, vascular risk factors, and disability [97, 98].

### Later-life risk factors

Despite LLD being associated with a range of medical comorbidities, few may directly contribute to depression pathogenesis. Age-related morbidities that are a focus of mechanistic models include inflammation, vascular disease, and neurodegeneration (Table 1).

**Table 1:** Support for etiological hypotheses of late-life depression.

	Inflammation	Vascular	Neurodegeneration
Clinical	• Neurovegetative symptoms (lethargy, reduced appetite) • Greater medical morbidity • Increased levels of pro-inflammatory cytokines; Decreased levels of anti-inflammatory cytokines • Associated with treatment resistance and poor antidepressant efficacy	• Dysphoria, anhedonia, apathy, psychomotor retardation, functional disability • Higher rates of vascular risk factors • Increased disability and mortality	• Apathy, subjective memory loss • AD pathology or development of dementia associated with poor antidepressant efficacy
Cognitive	• Executive dysfunction, slowed processing and motor speed, reduced memory	• Executive dysfunction, reduced processing speed and visuospatial skills, retrieval-based memory deficits	• Depression co-occurring with dementia worsens cognitive performance • Amnestic cognitive profile (often, but not always)
Imaging	• Peripheral inflammatory markers linked with: • altered fronto-subcortical activation • gray and white matter volume loss • Higher markers of central inflammation found in anterior cingulate and temporal cortices	• WMH volumes increase over time • Higher WMH volumes worsen cognitive outcomes • Persistent depressive symptoms exhibit greater change in WMH volume over time	• LLD with greater beta-amyloid deposition have: • greater temporal lobe volume loss • lower functional connectivity in fronto-subcortical regions • functional DMN alterations
Negative findings	• When excluding comorbid medical conditions, studies do not show relationship between depression and inflammatory cytokines	• Inconsistent findings between LLD, vascular burden, and antidepressant response.	• Some report less beta-amyloid deposition in LLD compared to controls • APOE ε4 does not clearly influence development of LLD

*AD* Alzheimer’s disease, *APOE* apolipoprotein E, *CSF* cerebrospinal fluid, *DMN* default mode network, *LLD* late-life depression, *WMH* white matter hyperintensities.

Table inspired by and adapted from Alexopoulos [164].

#### Inflammation

The inflammation hypothesis proposes that immune dysregulation influences vulnerability to and the development of LLD [99]. Neurovegetative depressive symptoms are akin to immune responses to infectious diseases including lethargy, cognitive slowing, and reduced appetite [100, 101]. In younger adults, elevated pro-inflammatory cytokines levels in response to psychological stress are associated with depressive symptoms [101] and induction of peripheral inflammation results in fatigue and worsening of mood [102, 103]. Depressed patients across the adult lifespan can exhibit elevated levels of pro-inflammatory cytokines including c-reactive protein (CRP), interleukin-6 (IL-6) and tumor necrosis factor (TNF) alpha and lower anti-inflammatory cytokine levels [104, 105]. Higher pro-inflammatory cytokine levels are associated with depression severity, suicide risk, and poor treatment response in adult and geriatric samples [105–107]. Pro-inflammatory cytokines are associated with worse function in executive processes, memory, and processing and motor speed [108].

Aging is itself associated with chronic, low-grade inflammation, dubbed “inflammaging” [109]. This process has multiple contributors, including immune system aging, mitochondrial changes, and gut microbiota [110, 111]. These changes may be secondary to common pro-inflammatory medical conditions that increase depression risk, including diabetes, cardiovascular disease, autoimmune disorders, rheumatoid arthritis, cirrhosis, and kidney disease [112–115]. These comorbidities may explain the observed relationship between depression and inflammation, as evidenced by a study that did not associate LLD with higher levels of central or peripheral inflammatory cytokines. However, this study employed rigorous entry criteria for medical comorbidities that excluded almost 95% of potentially eligible participants [116]. This raises issues about its generalizability and highlights the extent of comorbidity between LLD and medical illnesses.

Although most work examines peripheral inflammatory markers, it remains relevant to brain function. The CNS was long considered to be immunoprotected due to the blood-brain barrier. However, immune responses via peripheral immune cell secretion of pro-inflammatory cytokines can convey the inflammatory response to brain microglia via humoral and neural pathways [117, 118]. Microglia can thus become activated by peripheral cytokines inducing a neuroinflammatory response [119]. Both aging and psychological stress further prime microglia toward an activated state, tilting the CNS toward a pro-inflammatory state [120, 121]. In the aged brain, activated microglia exhibit an exaggerated response to pro-inflammatory cytokines, inducing oxidative stress and delayed clearance of neurotoxic molecules, resulting in disrupted neuronal function, impaired neurogenesis, and neural degeneration [119, 120]. Central inflammation further affects multiple neurotransmitter systems, contributing to reduced serotonin synthesis via induction of indoleamine 2,3-dioxygenase [100], glutamate system dysregulation [122–124], and altered dopamine synthesis, binding, and reuptake [37, 125–128].

While less examined in LLD, chronic inflammation induces an altered cellular environment in the brain parenchyma capable of modulating neural circuits and influencing depressive behavior [15]. Pro-inflammatory cytokines including CRP and IL-6 are associated with global gray matter and white matter loss [129]. A meta-analysis including participants across the lifespan associated peripheral inflammatory markers with altered activation of the prefrontal cortex, insula, striatum, amygdala, hippocampus, and various subcortical regions [130]. These regions overlap with the DMN, ASN, limbic, and corticostriatal networks. Studies of individuals with depression and suicide attempts report increased microglial activation in the anterior cingulate cortex (ACC), a key hub of both the ASN and CCN, in individuals with depression and suicide attempts [131, 132]. Experimental induction of acute inflammation similarly alters glutamate metabolism in the ACC and basal ganglia [124].

Treatment implications of pro-inflammatory processes are unclear. Work in midlife suggests that low-grade inflammation may decrease antidepressant efficacy [133–136]. However, successful antidepressant treatment can decrease pro-inflammatory cytokine levels [137, 138] Conversely, antidepressant augmentation with anti-inflammatory agents may reduce depressive symptom severity and improve treatment outcomes [139, 140]. If existing trials are supported, such interventions may most benefit individuals with higher inflammatory cytokine levels, such has been seen with infliximab, a TNF-alpha antagonist [141, 142]. Inflammation could identify a distinct phenotype [37, 143] who would benefit from anti-inflammatory treatments.

#### Vascular disease

Cerebrovascular system changes are common with normal aging [144]. Cerebral small vessel disease (CSVD) describes leakage of the blood-brain barrier and dysfunction of cerebral autoregulation, neurovascular coupling, and capillary blood flow [145]. This causes cerebral perfusion deficits and hypoxia, triggering inflammation and neuronal death. Vascular risk factors including hypertension, atherosclerosis, and diabetes contribute to CSVD and result in the thickening of the penetrating small arteries, fibrosis of the vessel wall, and depletion of vascular smooth muscle cells.

The “vascular depression hypothesis” [146, 147] posits that CSVD may predispose, precipitate, or perpetuate LLD. This process likely begins in adulthood, as midlife cerebrovascular burden predicts increased depression severity over time [148]. The neuroradiological manifestation of “vascular depression” includes white matter hyperintensities (WMHs) on T2-weighted MRI, subcortical lacunes, and microbleeds [147, 149]. Mechanistically, WMHs may contribute to a disconnection syndrome where damage to communicating cortical-subcortical pathways involved in mood regulation and cognitive processes increases LLD vulnerability [78, 150, 151]. Further supporting a mechanistic role, meta-analyses have shown that late-onset depression show significantly greater WMH burden in late-onset LLD than in early-onset LLD [152, 153]. The extent of ischemic injury extends beyond visible WMHs, as vascular risk factors compromise microstructural integrity in normal-appearing white matter [154, 155]. Location of WMHs and microstructural changes may be critical, as LLD is associated with damage to the cingulum bundle, uncinate fasciculus, and superior longitudinal fasciculus [156–159].

These processes influence longer-term negative outcomes. Even without depression, vascular changes are associated with cognitive deficits (including executive dysfunction and retrieval-based memory deficits) and altered emotion processing [160]. Cross-sectionally, depressive symptoms with greater WMH volumes worsen cognitive outcomes in the early stages of CSVD [161]. Greater WMH volume in LLD contributes to greater longitudinal decline in executive functions and increased risk for dementia [162]. Individuals with persistent depressive symptoms similarly exhibit greater increases in WMH volume over time [163]. Greater vascular burden may be associated with poorer response to pharmacological and nonpharmacological treatments [158, 164–166], although this relationship for medication response is somewhat weak [49, 78]. The higher vascular burden is further associated with greater disability [167], gait and other motor deficits [168], and frailty [169]. Depression can also worsen cardiovascular and cerebrovascular disease outcomes [170, 171], suggesting a bidirectional relationship.

#### Neurodegeneration

Aging is the strongest risk factor for dementia [172], a collective term for cognitive impairment negatively affecting independent functional activities. Alzheimer’s disease (AD), the most common dementia, is characterized by abnormal accumulation of beta-amyloid plaques and tau tangles in the brain. Early AD typically affects memory centers, including the entorhinal cortex and hippocampus. With disease progression, neuropathology spreads to frontal and parietal regions and affects language, executive abilities, and social behaviors.

Depression and dementia exhibit a bidirectional relationship. Depression in mid-to-late life increases risk for AD and all-cause dementia [14, 173, 174]. Depression can also be a precursor to or symptom of dementia, with prevalence rates ranging from 17 to 56% across all stages of AD [175]. Depression co-occurring with dementia worsens cognitive performance beyond what would be expected based on neuropathology alone [176]. Dementia risk may be highest in individuals exhibiting persistent or worsening depressive symptoms over time [177].

Such observations led to work searching for common genetic factors. While the apolipoprotein E (APOE) ε4 allele significantly increases risk for AD, it does not clearly influence the development of LLD [178, 179]. A genome-wide association study found that depression had a causal role in AD through Mendelian randomization, but there was no evidence for a causal role of AD on depression [180]. That study identified 53 brain transcripts and proteins regulated by the depression GWAS signals that also were associated with rates of cognitive decline over time [180].

The “amyloid hypothesis of LLD” [181] is supported by observations of increased beta-amyloid deposition in older adults with a depression history [182] and in LLD patients exhibiting a cognitive profile suggestive of amnestic Mild Cognitive Impairment [183]. Individuals with LLD exhibiting greater beta-amyloid deposition show greater volume loss in the temporal lobe, lower functional connectivity in fronto-subcortical regions, and greater functional alterations in the DMN [184, 185]. These findings are not universal, and the Alzheimer’s Disease Neuroimaging Initiative depression group reported less beta-amyloid deposition in LLD compared to a control group [186]. While beta-amyloid deposition was associated with worse memory performance in that study, the association between amyloid and cognitive performance did not differ between diagnostic groups. More recent work has focused on tau pathology as others report that individuals with elevated tau, but not amyloid, are twice as likely to be depressed [187].

Poorer cognitive performance, comorbid dementia, and AD pathology are associated with poorer prognosis for response to antidepressant medications. Both poorer cognitive performance, particularly executive dysfunction or slowed processing speed, and dementia are associated with poorer responses to antidepressant medications [12, 165, 188, 189]. Similarly, higher levels of beta-amyloid deposition, particularly in the temporal lobe, are associated with poor response or treatment resistance to antidepressant medications, even in cognitively intact elders [190, 191]. Alternative treatment approaches are not entirely clear, although some individuals with cognitive impairment may benefit from nonpharmacological interventions such as computerized cognitive training [192].

Depression is not unique to a single neuropathological process [176]. Beyond AD, it occurs in context of alpha-synuclein, a constituent of Lewy bodies and the pathological hallmark of synucleinopathies, including Parkinson’s disease (PD), dementia with Lewy bodies, and multiple system atrophy. Depression is a common non-motor symptom of PD [193] and depressed individuals exhibit a 2.2-fold increase in risk of subsequent parkinsonism [194]. There is a positive association between alpha-synuclein messenger RNA expression levels and depression severity [195], while levels of CSF alpha-synuclein may mediate associations between LLD, markers of synaptic dysfunction, and memory ability [196].

## Depression and accelerated aging

Both vascular [170, 171] and neurodegenerative processes [14, 173, 174] occur more frequently or more severely in LLD. These may represent bidirectional relationships, where depressive episodes may both contribute to and result from accelerated aging. Biological aging is an inevitable process at molecular, cellular, and organ levels reducing a system’s reparative or regenerative potential [197]. “Accelerated aging” is when biological aging occurs more rapidly than expected, resulting in biological characteristics that are more severe than would be expected based on chronological age [198]. In the brain, this may include ventricular enlargement, cerebrovascular injury, or gray matter atrophy. For this review, we distinguish accelerated aging from “pathological brain aging”, characterized by neurodegenerative processes involving amyloid, tau, or other abnormal protein deposition.

Accelerated aging is observed in multiple neuropsychiatric disorders and quantified using a range of markers including telomere length, oxidative stress markers, epigenetic markers, physiological functioning, and neuroimaging [199–201]. In adult MDD, accelerated aging is observed on molecular and cellular markers, including reduced telomere length, epigenetic aging, and metabolomic aging [201, 202]. Accelerated aging in MDD is further observed on both structural and functional neuroimaging, where depressed individuals appear on average 1–2 years older than nondepressed cohorts [203, 204]. In the largest of these studies, the difference between calculated brain age and chronological age was independently replicated [205] and not associated with age of depression onset, recurrence, or remission [204].

An accelerated aging hypothesis of LLD implies a bidirectional process [206]. Depressed older adults exhibit an advanced biological age on a multibiomarker index of metabolic and inflammatory measures [207, 208] and on structural MRI, where they appear approximately 4 years older than nondepressed individuals [198]. This accelerated brain aging is further associated with disability and cognitive performance [198], with depression severity moderating the relationship between brain age and some cognitive measures. As the difference between calculated brain age and chronological age differs between midlife adult depressed samples and LLD [198, 203, 204, 209], depression may be associated with an altered trajectory of biological aging [210].

Accelerated aging also influences physical function and contributes to physical frailty. Frailty is characterized by deficits in strength and mobility, decreased physical activity, and reduced energy capacity that results from dysregulation in metabolic, musculoskeletal, and stress-response systems [211, 212]. Frailty is common, bidirectionally associated with LLD, and associated with increased mortality and poor antidepressant treatment responses [213–215]. Frailty may be an outcome of depression, as depression is associated with worsening trajectories in functional status, including reduced walking speed and hand strength [216].

**Fig. 3:**
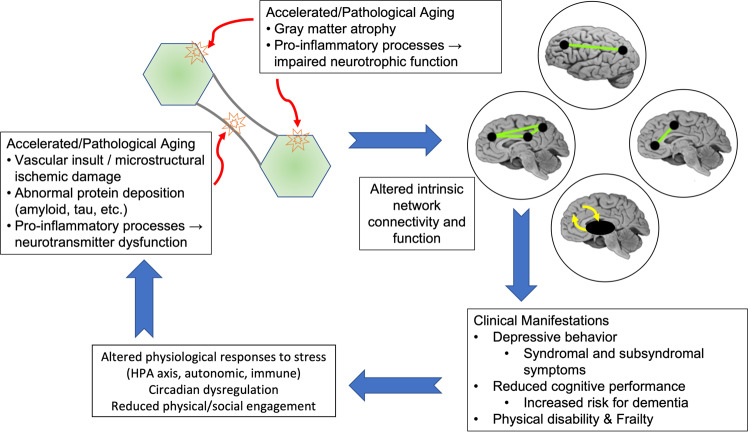
Accelerated aging hypothesis of late-life depression. Aging processes such as inflammation, vascular disease, or pathological neurodegeneration impair neurotrophic function and contribute to both gray matter atrophy and impairment of white matter microstructure. These changes in turn alter function of key intrinsic networks, leading to the clinical manifestations of late-life depression. In turn, repeated depressive episodes result in altered or sustained physiological responses increasing allostatic load. These effects may then further accelerate biological aging processes, shifting an individual further away from normative aging.

The model of a bidirectional relationship between depression and accelerated and pathological aging (Fig. 3) may start with age-related changes increasing vulnerability to depression. Individuals experiencing accelerated biological aging, operationalized as advanced medical morbidity, cerebrovascular pathology, pro-inflammatory processes, or pathological aging, such as increasing amyloid or tau burden, are at higher risk for LLD. Such etiological factors can disrupt the structure, function, and homeostasis of key intrinsic functional networks implicated in depression [78, 164]. These etiological factors may all occur to varying extents in the same individual, each challenging functional network homeostasis. As the underlying systems become more dysregulated or as pathologies progress, and networks experience greater homeostatic imbalance and impairment in function, the clinical presentation of the depressive syndrome emerges (Fig. 1) [15]. It is possible that the predominant underlying pathology and what brain networks are most affected influence the clinical presentation and phenotype [37, 217].

Depressive episodes may also accelerate biological aging (Fig. 2). One potential mechanism involves the altered neural and physiological responses to stress observed in depression. Such altered responses are observed across a range of systems, including altered function of brain regions involved in emotion processing, autonomic reactivity, HPA axis function, and dysregulation of the immune system or circadian processes [78, 99, 218–224]. Normally, these processes are meant to facilitate allostasis, the body’s ability to respond to environmental challenges and maintain normal functioning. However, with repeated stressors and depressive episodes, these responses contribute to increased allostatic load, the wear-and-tear resulting from stress and the dysregulation of processes meant to maintain homeostasis [15, 225]. Such canonical stress-response systems interact and over time contribute to accelerated aging, leading to regional brain atrophy, development of cerebrovascular pathology, and reduction of neurotrophic function [226–228]. This hypothesis is supported by longitudinal studies associating persistent or recurrent depression with greater increases in WMH volume and greater hippocampal volume decline [163, 229]. Other mechanisms explaining this relationship are possible, including shared genetic vulnerabilities [180].

Such bidirectional relationships have long-term implications. Accelerated or pathological aging also contribute to impaired cognitive performance [198, 230], increased risk for dementia [14], and risk of physical disability, sensory function loss, and frailty [230]. Accelerated brain aging may be associated with higher risk for depression relapse after achieving remission [15]. Even if the initial antidepressant treatment were successful, progressive aging may further challenge functional networks and result in a return of depressive symptoms [15].

## Updates on established somatic treatments

Currently, treatment decisions for patients with LLD are guided more by clinical history and patient preference than potential biological causal factors. Robust blinded clinical trials data for LLD are scant and clinical guidelines tend to derive from expert opinion or are extrapolated from data in younger populations [231].

### Antidepressant medications

Antidepressants are more effective than placebo in the treatment of LLD, although the response rate is lower for older than younger adults [232–234]. However, while antidepressants alter DMN and CCN connectivity [235], age of initial onset does not influence antidepressant medication response rates, although early-onset patients may respond more slowly [236, 237]. Antidepressants remain beneficial, with a number needed to treat (NNT) for an antidepressant response being 6.7 (95% CI, 4.8–10) [234, 238]. As in younger adults, augmentation strategies in LLD are more efficacious than strategies involving a switch to a different antidepressant [239]. Methylphenidate augmentation of an SSRI is superior to monotherapy with either agent alone [240]. Augmentation with lithium, bupropion, or aripiprazole in patients who did not respond to monotherapy can be well-tolerated and improve depressive symptoms [239, 241, 242]. Despite clear benefits of augmentation, the likelihood of achieving remission decreases with increasing number of failed antidepressant trials within the current episode [243].

Few studies examine outcomes of the N-methyl-d-aspartate (NMDA) receptor channel inhibitors ketamine and esketamine in LLD. Both a small, randomized trial of subcutaneously administered ketamine and larger open-label study of intravenous ketamine improved depression severity in older adults with treatment-resistant depression [244, 245]. Intravenous ketamine resulted in a remission rate of 12.8% for older individuals, which is comparable to remission rates seen in patients progressing to later stages of the STAR*D study [245, 246]. A randomized trial of esketamine in treatment-resistant LLD resulted in a comparable remission rate of 17.3%, with a NNT of 10, although that study did not detect a statistically significant difference in their primary endpoint [247]. Secondary analyses suggested that participants with an earlier life onset of depression or who were less than 75 years of age exhibited greater improvement [247]. Both ketamine and esketamine were well-tolerated, with common side effects including dizziness, dissociative symptoms, fatigue, and transiently elevated blood pressure [245, 247].

### Electroconvulsive therapy (ECT)

ECT continues to be used for severe and treatment-resistant LLD. In LLD, ECT exhibits remission rates between 70 and 90%, although rates in community samples may be lower [248]. Individuals with late-onset depression tend to respond better to ECT than individuals with early-life depression onset [249], which may be related to illness chronicity or recurrence in the early-onset group. However, strong remission rates should be balanced by high relapse rates after the initial ECT course, with 40–50% of patients relapsing within 6 months [250]. Cognitive side effects in older adults tend to be limited and transient [251].

Recent work has refined ECT to improve tolerability while preserving efficacy. This includes administering ECT using right unilateral electrode placement with ultrabrief pulse width stimuli, an approach with fewer cognitive side effects [252]. When combined with venlafaxine in LLD, this results in remission rates of 61% and response rates of 70% [253]. Unilateral brief pulse ECT combined with venlafaxine only modestly affects cognitive performance, specifically letter fluency and cognitive flexibility [254]. This study also included a 24-week continuation phase, where participants were randomized to either medication only (venlafaxine plus lithium) or venlafaxine plus continuation ECT, administered weekly for the first month with additional sessions as needed. Continuation ECT resulted in lower levels of depression severity at study endpoint than medication only [255] and better quality of life [256].

### Transcranial magnetic stimulation (TMS)

Repetitive TMS (rTMS) uses a pulsed magnetic field to induce a local electrical field on the brain’s surface, stimulating cortical pathways. rTMS treatment of depression typically targets the DLPFC, with the best-studied techniques including unilateral high-frequency left-sided (HFL), unilateral low-frequency right-sided (LFR), or sequential bilateral treatment of LFR followed by HFL [257]. Parallel work supports that targeting the DLPFC modulates functional connectivity within and between the DMN and CCN, with clinical benefit deriving from modulation of subgenual cingulate cortex connectivity [258]. While many randomized trials support rTMS efficacy, few have been conducted in LLD [259, 260]. However, rTMS is well-tolerated in older adults [261], and LLD trials generally support the efficacy of HFL rTMS, with bilateral treatment being more efficacious in treatment-resistant patients [257].

Recent work has modified rTMS to improve outcomes and reduce burden. A sham-controlled trial in LLD that examined bilateral deep rTMS reported efficacy and good tolerability when administered over four weeks [262]. This deep TMS approach addressed concerns that age-associated atrophy may contribute to poor treatment responses by increasing the distance between the scalp and cortex [263], however it requires longer administration sessions. More recent work in LLD compared rTMS to theta-burst stimulation (TBS), a bilateral approach that reduces session administration from 47 min for rTMS to 4 min for TBS. This randomized trial established non-inferiority of TBS, with comparable reductions in depression severity between groups [264].

## Resilience factors: opportunities for intervention

Although these treatments are effective, benefit depends on continued treatment. If pharmacotherapy or neuromodulation stops, the risk of recurrence can be high [18, 250, 253]. This risk may be reduced through interventions that target vulnerability factors to depression and strengthen resiliency (Table 2).

**Table 2:** Resilience factors influencing depressive episode risk in later life.

Domain	Factors	Resilience correlates	Depression vulnerability correlates
Trait-like factors	Temperament	Positive emotionality	Greater harm avoidance
	Personality	Extroversion, conscientiousness	Neuroticism
Psychological factors	Beliefs	Self-esteem, self-efficacy, mastery, sense of purpose	Internalized self-blame or stigma
	Coping	Active or accommodative coping	Avoidance or passive coping
Social factors	Social support	Social engagement	Social withdrawal, loneliness
	Altruism	Formal volunteering	Social role absences
Cognitive factors	Cognitive reserve	Maintained cognitive performance	Poorer executive function and processing speed
Physical factors	Physical activity	Exercise, regularly active	Sedentary lifestyle
	Sensory function	Sustained or corrected vision and hearing	Impaired sensory function
	Healthy diet	Good nutrition	Poor nutrition, substance abuse
	Healthy sleep	Regular sleep patterns	Disrupted, irregular sleep

Correlates of factors that may contribute to resilience from depression, or vulnerability to depression. Some factors are modifiable (psychological factors, social factors, and lifestyle factors) while others are not (trait-like factors). Some depression correlates may both increase the risk of depression and also be an outcome of depression. Table inspired by and adapted from Laird et al. [267] and Andreescu et al. [15].

Resilience is broadly defined as the capacity to maintain or regain psychological well-being despite challenges, or the adaptive maintenance of homeostasis despite adversity [265–267]. Resilience is a multidimensional, dynamic process influenced by both internal factors and external resources. Resilience factors may decrease the risk of a depressive episode, reduce severity or duration of that episode, or increase likelihood of recovery [267, 268]. If depression contributes to accelerated biological aging, then bolstering such resilience factors and reducing the frequency or duration of depressive episodes could hypothetically shift the biological aging process towards a more normal trajectory. We discuss resilience factors and their corresponding vulnerability factors (Table 2) in context of treatments.

### Psychological factors: role for brief psychotherapy

While factors such as temperament and personality may be challenging to target with brief therapy, progress can improve negative beliefs and coping strategies. Individuals’ beliefs about themselves and their environment influence how they cope with stressors or challenges. Depression risk is associated with lower self-esteem, anxiety sensitivity, and an external locus of control (i.e., a feeling that one cannot influence outcomes in one’s life) [269]. The converse of these beliefs contribute to resilience, as does a sense of purpose and grit, defined as perseverance in achieving goals despite setbacks [267, 270–272]. Greater self-efficacy enhances individuals’ ability to flexibly apply coping strategies, including active coping strategies that directly address the stressor, or accommodative strategies involving adaptation to the stressor. Such a flexible approach improves mental health outcomes and reduces depression [273, 274].

#### Evidence for psychotherapy

Psychological factors addressing vulnerability or promoting resilience may be particularly amenable to psychotherapy. Evidence-based treatments in LLD include cognitive-behavioral, problem-solving, interpersonal, and life-review therapies [275, 276]. Such therapies influence functional network connectivity, such as cognitive-behavioral therapy increasing connectivity between the amygdala and CCN [277]. Meta-analyses in LLD support that psychotherapy is quite effective, with a NNT of 3 [278]. More recently developed, “Engage” therapy targets neurobiologically-informed processes in LLD using streamlined behavioral techniques that can be effectively applied in the community [279, 280].

### Social factors: opportunities for engagement

Aging adults tend to maintain close social partners but have fewer peripheral social contacts [281]. In contrast, larger objective social network size and greater perceived social support protects against LLD and predicts a better response to depression interventions [282–284]. Such benefits may occur through mechanisms including emotional support, tangible assistance (instrumental support), or opportunities for pleasurable activities [285]. Recent work has focused on loneliness, or perceived social isolation that is distinct from having fewer objective social contacts. Loneliness is bidirectionally associated with a host of negative outcomes, including depression, poor physical health, cognitive and functional decline, and mortality [286–288]. Loneliness may be a neuropsychiatric manifestation of preclinical AD, as it is associated with an elevated dementia risk and higher levels of amyloid and tau pathology [289–291].

#### Evidence for targeting social connectedness

Few intervention studies directly target social factors in depression [292]. Group therapy benefits LLD [293] but does not typically focus on social connectedness. Recent novel work has examined remote, layperson-delivered interventions intended to improve social connectedness and reduce loneliness in younger and older adults. Although not directly targeting individuals with a depression diagnosis, they reduced depressive and anxiety symptoms [294–297]. Modifying existing psychotherapies to target social disconnection may also reduce suicide risk [294].

### Cognitive factors: role for cognitive training

As previously discussed, LLD is associated with cognitive changes in executive functioning, processing speed, and episodic memory [8–11]. However, not all individuals with LLD have cognitive difficulties. There appear to be separate cognitive phenotypes within LLD: “High Normal”, “Reduced Normal” (with a relative weakness in episodic recall), and “Low Executive Functions” [298]. The “High Normal” phenotype maintained cognitive performance despite similar levels of depression severity as the other phenotypes. They also had higher levels of education and less vascular risk factors, suggesting that cognitive reserve and vascular health may contribute to cognitive resilience in LLD [299]. Identifying cognitive difficulties unique to the depressed individual allows for the prescription of personalized cognitive training interventions.

#### Evidence for benefits of cognitive training

For computerized cognitive training to work, it must have an adequate duration and be appropriately intense or difficult [300]. Evidence for the potential benefit of neurobiologically-informed computerized cognitive training in LLD comes from the approaches by Morimoto and others that are optimized to treat LLD with executive dysfunction [301]. They demonstrate that by targeting the underlying deficient neural circuitry and associated cognitive deficits in LLD, cognitive functions and depressive symptoms improve, benefits transfer to non-trained domains such as memory, and there are positive changes to underlying neural structural-functional connections [192, 300, 302]. Targeted cognitive training appears to modulate network functional connectivity in the DMN and CCN [303, 304].

Interventions targeting memory deficits (such as the Mayo Clinic’s Healthy Action to Benefit Independence and Thinking (HABIT) program) [305] benefit Mild Cognitive Impairment both with in-person and virtual platforms. While this intervention has not been conducted in LLD, the approach could translate to other populations with primary memory issues. Similarly, processing speed training shows benefit for up to 10-years in nondepressed older adults [306] and may be particularly favorable for LLD characterized by predominant cognitive and motor slowing. Augmenting cognitive training with neuromodulation approaches or other nonpharmacological treatments may provide additional benefit but need study in LLD [307].

### Physical disability: need for sustainable movement-based interventions

Motor deficits are common with aging, including slowing, coordination deficits, and balance difficulties [308, 309] and they contribute to falls, disability, and mortality [310–314]. Depressed older adults are at increased risk for these motor problems [310, 314, 315]. This may be due to common contributors or comorbidities, such as vascular disease or other brain pathology [316, 317]. However, by increasing sedentary behavior and isolation, depression may also hasten muscle atrophy, deconditioning, and frailty [37, 213–215].

#### Evidence for movement-based interventions

Structured physical exercise benefits depression symptoms across the adult lifespan [318], including moderately benefitting older adults [319, 320]. It has positive benefits on hippocampal volume [321], may modulate connectivity between key DMN and CCN regions [322, 323], and also augments the response to antidepressant medications [324]. Physical activity improves global cognitive function in unimpaired elders and benefits cognitive domains sensitive to aging, including attention, executive function, and memory [325, 326]. Similarly, physical exercise, particularly aerobic activity, may reduce the risk of dementia and benefit older adults with existing cognitive impairment or dementia [327, 328]. Interestingly, recent work suggests that mind-body therapies that combine movement-based approaches with mindfulness, such as yoga or tai chi, may be superior to conventional exercise for mood and cognitive outcomes [267, 329]. However, questions remain about optimal practices needed to obtain such benefit, including frequency, intensity, duration, and type of exercises [303]. Strategies to facilitate initiation and maintenance of exercise in community-based elders are sorely needed, particularly for individuals with chronic pain or disabilities that limit physical function.

### Sensory impairment: can improving sensory function help?

Sensory impairment, including vision and hearing loss, is also common in later life. Uncorrected hearing loss and vision loss are associated with greater depressive symptom severity and increased risk of developing LLD [217, 330–335], particularly for dual sensory loss affecting both vision and hearing [336]. Several hypotheses could explain these relationships, including how sensory loss limits social activities, leading to isolation and worsening psychological health [332, 337]. This may not account for the entire relationship, as sensory loss is further associated with physical decline [338], cognitive decline, and risk of dementia [335, 339].

#### Evidence for interventions improving sensory function

Optimizing sensory function benefits depressive symptoms and reduces depression risk. For impaired vision, improving residual vision and self-management programs can reduce depressive symptoms [340, 341]. Integration of psychotherapy techniques, such as behavioral activation can prevent depression in high-risk patients with macular degeneration [342]. A similar benefit is seen in preliminary clinical trials demonstrating that hearing aids may benefit depressive symptoms, quality of life, and cognitive performance in older adults [343–345].

## Conclusions

We repeatedly describe bidirectional relationships between LLD and lifespan factors such as aging, inflammation, vascular disease, and more. These reciprocal relationships in essence describe positive feedback loops. In the absence of counterbalancing forces, such feedback loops can spiral out of control. Reframed in the allostatic framework, maintaining stability requires adaptive regulation and resiliency.

This dynamic nature of allostatic processes contributes to LLD heterogeneity. Vulnerability to developing depressive episodes results from accumulated factors, many of which have an initial onset earlier in life. Other vulnerability factors are unique to later life and may contribute to a new diagnosis of depression or a relapse of symptoms in previously remitted individuals. We propose that these vulnerability factors (Fig. 1) have negative effects on functional brain networks that predispose networks towards a state of fragility and instability.

Etiological heterogeneity creates challenges for understanding both LLD’s neurobiology and variability in treatment responses. It also creates opportunities to use this heterogeneity to probe specific mechanisms and guide focused, personalized treatment approaches. Such examples include examining dopaminergic system influences on LLD in patients with psychomotor slowing, testing anti-inflammatory medications in patients with elevated inflammation, or using targeted cognitive training to treat patients with LLD and executive dysfunction. While no single treatment will improve symptoms in all patients with LLD, combining established treatments such as pharmacotherapy, psychotherapy, and neuromodulation alongside personalized interventions that bolster resilience or address comorbid disability may improve outcomes for otherwise treatment-resistant patients.
